# Digestive Agents in Inflammatory Rheumatism

**Published:** 1881-02-20

**Authors:** S. V. Swentningen

**Affiliations:** Ind.


### DIGESTIVE AGENTS IN INFLAMMATORY RHEUMATISM.

BY S. V. SWENTNINGEN, M. D., IND.

It is not my purpose to attempt an exhaustive treatise upon the history, pathology, etiology and therapeutics of acute articular rheumatism. My aim is, rather, to induce the profession to make a trial of the remedy I propose to introduce, inasmuch as my own data are insufficient to warrant any positive conclusions as to the confidence it should merit in the treatment of this disease, and as a considerable period of time may elapse before another case of it will occur in my practice.

The therapeutics of rheumatic fever is, in my opinion, as unsatisfactory as its etiology and pathology; a fact which seems to justify the practitioner in resorting to experiment in its treatment. This was the light in which I considered the subject when called recently to treat a case of the disease under consideration.

Heretofore, in the management of this malady, I have pursued the plan of treatment generally recommended by our authorities; the administration of alkalies, salicylic acid, salines, quinine, tincture of iron, colchicum, lemon-juice, leeches, wrapping the joints in cotton, etc., etc.

The results in my hands of this routine of treatment have been so universally unsuccessful, that I finally directed my attention only to the relief of pain and the protection of the heart, and waited patiently for the expiration of the "six weeks" (or eight or ten, as the case may have been), for convalescence to become established.

In a typical case, however, which I am about to relate, after three days of treatment by the old plan, I decided to forsake it and employ some new agent which, to the best of my knowledge, had never been prescribed.

On the first of the present month (Dec., 1880), I was called to see George Humphrey, aged 15 years. I found him bathed in an acid perspiation, and screaming with pain, which migrated from ankle to ankle, knee to knee, wrist to wrist, elbow to elbow, and from his righ hip to his right shoulder. Temperature 104° F., tongue covered with a peculiar pasty, creamy coat, pulse beating at the rate of 140, rather soft and compressible. Urine somewhat scant in quanity, highly colored and acid in its reaction. The slightest touch upon the radial artery caused him pain, and it was with great difficulty that he could be moved at all. In short, it was a characteristic case of the complaint in question, the cause of which he attributed to sleeping in a damp bed away from home.

I immediately directed the following treatment:

℞ Acid salicyl., ..........................................

Quin. sulph.............................................. aa ʒ ss

Opii pulv.,.............................................. grs. vj

Morph. sulph.,........................................... gr. j. M.

Ft. capsulæ No. vj. div. et.

Sig. One capsule every four hours.

℞ Potass. acet.,.................................................. ʒ iij

Sprts. æth. nit.,.............................................. f. ℥ j

Tr. aconit. rad.,............................................ gtt. xv

Syr. ipecac.,................................................. f. ʒ ij

Liq. am. citrat , q.s.ad.................................. f. ℥ iv.

Ft. sol., et

Sig. A tablespoonful two hours after each capsule.

℞ Sodæ et potass. tart.,........................................ ℥ ij

Sig. Add to a glass of water and give one-third of the solution every two hours, until it operates freely on the bowels.

Pursuing the treatment for a period of seventy-two hours, and noticing no improvement in his condition, other than that which might be naturally expected from the effect of the opiates, which, only to a moderate degree, however, mitigated the pain, I began to cast about, mentally, for some other remedy.

It was necessary, of course, to frame some theory as a basis for the selection of the new agent, and jumping at the conclusion that the predisposing cause of the malady was some unknown error in disgestion, either gastric or intestinal, or both, the idea that lactopeptine or ingluvin might be of some benefit, at once suggested itself. I decided to give both, and accordingly left the following prescriptions :—

℞ Lactopeptine,..................................................

Ingluvin,...................................................... aa ʒ ss

Opii pulv., ................................................... grs. viij

Morph. sulph.,.............................................. gr. j. M

Ft. capsulæ No. vj. et

Sig. One capsule every four hours.

Not wishing to discard entirely the usual time-honored treatment, sanctioned by so many able authors. I alternated each dose of the foregoing prescription with a tablespoonful of the following :—

℞ Potass. iodid.,.............................................. ʒ j

Potass. bicarb.,............................................... ʒ iij

Aquæ destil.,............................................... f. ʒ iij

Tr. colch. sem.,............................................ f. ʒ ij

Tr. gent. co., .............................................. f. ʒ iij

Syr. simp., q.s.ad......................................... f. ʒ iv M.

Ft. sol., et.

Sig. A tablespoonful two hours after each capsule.

Upon my next visit, which was made after the expiration of twenty-four hours, I was very much surprised, indeed, to notice such a well-marked general improvement in the condition of my patient. His parents were likewise happily astonished, for I had prepared them to expect a six weeks' siege of the attack. The pain, swelling and temperature had decidedly subsided, and he could now move a number of muscles with comparative ease.

Fearing that his apparent convalescence was a mere respite, but of short duration, and that the enemy would ere long make a fresh and more vigorous attack, I directed the prescriptions to be renewed and taken as before.

Upon the next visit, twenty-four hours subsequent, I discovered that my patient had arisen from his bed, dressed himself, and walked down stairs into the sitting room, where I found him feeling quite comfortable; convalescence having rapidly progressed unchecked. Of course, all these efforts upon his part were not made without the assistance of attendants. From this period up to the present his condition has been one of continued improvement, and he has now apparently regained his former health.

Now, if it were not for the fact that the latter prescription figured prominently and faithfully in the treatment of three cases which I had under care at one and the same time, in the spring of 1878 (and which pursued the "even tenor of their way" for six, eight, and nine weeks, quite unimpressed with my best efforts at medication), I should be inclined to attribute to it the success achieved in the case just related. I failed to detect any particular good that it acomplished in the cases referred to, and hence feel justified in the present case in giving the credit to the lactopeptine, or inguluvin, or both.

But this is simply an individual case in my own individual experience. It must stand the test of collective cases, and for his purpose I present it to the profession.—*Medical and Surgical Reporter.*

